# Predicting the need for cerebrospinal fluid shunt implantation after spontaneous intracerebral hemorrhage: a challenging task

**DOI:** 10.3389/fneur.2023.1255477

**Published:** 2023-12-07

**Authors:** Farjad Khalaveh, Vitalij Zeiser, Anna Cho, Sophie Schmelzer, Andrea Reinprecht, Johannes Herta, Karl Roessler, Christian Dorfer

**Affiliations:** Department of Neurosurgery, Medical University of Vienna, Vienna, Austria

**Keywords:** cerebral hemorrhage, cerebrospinal fluid shunt, hydrocephalus, intraventricular hemorrhage, shunt dependency

## Abstract

**Objectives:**

The development of persistent hydrocephalus in patients after spontaneous intracerebral hemorrhage (ICH) is still poorly understood, and many variables predicting the need for a cerebrospinal fluid (CSF)-shunt have been described in the literature with varying results. The aim of this study is to find predictive factors for shunt dependency.

**Methods:**

We performed a retrospective, single-center study of 99 neurosurgically treated patients with spontaneous ICH. Variables, including age, Glasgow Coma Scale (GCS), intraventricular hemorrhage (IVH), location of hemorrhage, acute hydrocephalus, and volumetric analysis of IVH, ICH, and intraventricular CSF were compared between patients with and without CSF-shunt implantation. Furthermore, receiver operating characteristics (ROC) for ICH, IVH, and intraventricular CSF volume parameters were calculated.

**Results:**

CSF-shunt implantation was performed significantly more often in patients after thalamic (*p* = 0.03) and cerebellar ICH (*p* = 0.04). Moreover, a lower ratio between the total hemorrhage volume and intraventricular CSF volume (*p* = 0.007), a higher IVH distribution in the third ventricle, and an acute hydrocephalus (*p* < 0.001) with an increased intraventricular CSF volume (*p* < 0.001) were associated with shunt dependency. Our ROC model demonstrated a sensitivity of 82% and a specificity of 65% to predict the necessity for a shunt at a cutoff value of 1.9 with an AUC of 0.835.

**Conclusion:**

Volumetric analysis of ICH, IVH, and intraventricular CSF may improve the prediction of CSF shunt implantation in patients with spontaneous ICH.

## Introduction

With an incidence of 246 out of 100,000 patients, spontaneous intracerebral hemorrhage (ICH) is the second most common cause of stroke (1, 2). In 45% of the cases, ICH leads to intraventricular hemorrhage (IVH) and is associated with the development of acute hydrocephalus with a worse outcome compared to those without IVH (3–5).

Placement of an external ventricular drain (EVD) is the most effective method to treat acute hydrocephalus and is typically necessary for 7.1–35.2% of the patients in the reported series (6–11). Which of these patients will eventually develop chronic hydrocephalus necessitating the placement of cerebrospinal fluid (CSF) shunt is an important and just as difficult question in daily practice. The availability of a prediction tool for later shunt dependency would help clinicians in guiding the treatment and care of patients with spontaneous ICH.

In general, the incidence of chronic hydrocephalus in patients with ICH is reported to be lower than that in patients with aneurysmal subarachnoid hemorrhage (SAH) (9, 12, 13). However, predictors of hydrocephalus, as they are available in SAH patients, are lacking (6–9). The thalamic location of the hemorrhage and increased intracranial pressure (ICP) have been shown to increase the likelihood of shunt dependency (6, 7). In contrast, an association between the volumes of the intraparenchymal and intraventricular hemorrhage and the development of chronic hydrocephalus could not be observed by these studies (6, 7, 10). Furthermore, one could assume that it is relevant whether a given blood volume is restricted to the lateral ventricles or distributed within the third or fourth ventricles to the development of chronic hydrocephalus. However, the results have not been convincing (9).

The decision to place an EVD is also multifactorial and certainly another influencing factor as patients with less objective signs of hydrocephalus may also undergo EVD placement just for the purpose of ICP monitoring. Some patients receive a one-sided EVD, and others receive bilateral EVDs. Together with the amount of daily CSF drainage all of the aforementioned factors may interplay and contribute to various extents to the need for a CSF-shunt placement in these patients.

To learn about these interactions, we thought to analyze our patient population with the aim to identify an applicable tool that helps us and others to better predict shunt dependency in patients after spontaneous ICH.

## Methods

The study protocol was approved by the local Ethics Committee of the Medical University of Vienna (EK 1055/2022; 15.02.2022). This study was performed in accordance with the ethical standards as laid down in the 1964 Declaration of Helsinki and its later amendments or comparable ethical standards. No formal consent was required for this type of study.

All patients, who underwent a neurosurgical treatment for a spontaneous ICH at our institution between the years 2008 and 2021, were retrospectively analyzed. ICH from neurovascular pathologies (e.g., intracranial aneurysms and arteriovenous malformations) or from tumors, and patients <18 years of age at the time of ICH were excluded from the study. We assessed the following variables: patient’s demographics, anatomical location of ICH, the intraventricular hemorrhage score (IVHS), date and type of surgery, duration of external ventricular drain (EVD) placement, amount of daily drained cerebrospinal fluid (CSF) during the total EVD treatment period, and various comorbidities such as hypertension, diabetes mellitus type 2 (DM2), and atrial fibrillation (14). At admission, the Glasgow coma scale (GCS) was assessed by a physician, and a cerebral computed tomography (CCT) scan was performed in all patients. The indication and type of surgical treatment were determined by the treating neurosurgeon on a case-by-case decision based on the following clinical and neuroradiological parameters.

### Neurosurgical treatment

Depending on the extent and location of the ICH, the following neurosurgical procedures were performed either alone or in combination.

### External ventricular drain (EVD) insertion

In cases of intraventricular involvement of the hemorrhage, suspicion of hydrocephalus and/or suspected ICP elevation or deterioration of the GCS < 8 with correlating neuroradiological findings, an EVD was inserted. Depending on the extent and pattern of the intraventricular hemorrhage and the decision of the neurosurgeon on call, patients received the EVD either unilaterally or bilaterally. As a standard, EVD insertion was performed at the Kocher’s point with a non-antibiotic coated ventricular catheter (Straight Ventricular Catheter F8, Integra^®^). The catheter was tunneled under the galea and secured by multiple sutures. All patients received at least a single dose of a periprocedural prophylactic antibiotic. According to our local weaning protocol, weaning of the EVD was started as soon as a gradual decrease of the drained CSF amount with decreasing CSF cell count could be observed, and CT scans looked accordingly. Consequently, the EVD drip chamber at the pole mount was increased by 5 cm/day if clinically justifiable. At 20 cm above the nose level, the EVD was clamped and subsequently removed if tolerated by the patient and in the absence of ventricular enlargement on a CT scan.

### Evacuation of ICH

Hemorrhage evacuation was performed in patients with space-occupying infratentorial cerebellar or supratentorial ICH with enough mass effect justifying clot removal. Although we did not stick to the surgical trial in lobar intracerebral hemorrhage (STICH) criteria over the study period in all cases, the indication for clot removal was generally based on these trial results. Preferentially, patients with large spontaneous ICH and intermediate GCS were candidates for hemorrhage evacuation (15–17). However, in the end, it was up to the discretion of the neurosurgeon on call whether or not the ICH was evacuated.

### Neuroradiological variables and volumetry

**Figure 1:**
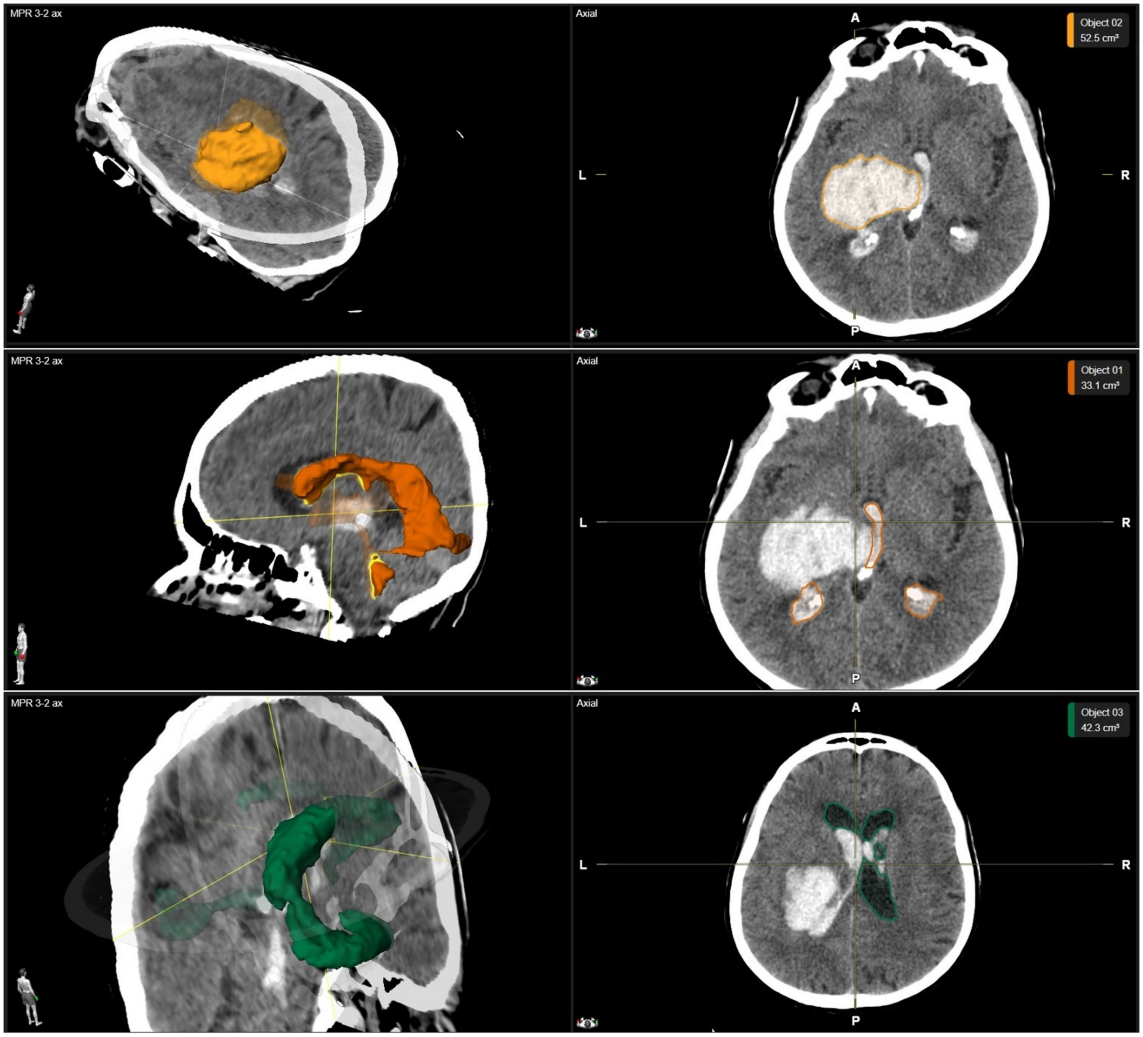
Example of volumetric data. The volumetric analysis of a thalamic ICH with IVH is shown in a three-dimensional reconstruction as well as axial planes of CCT images. Above row: The volume of a thalamic ICH is highlighted in yellow. Middle row: The volume of IVH is highlighted in orange. Below row: The volume of intraventricular CSF is highlighted in green. CCT, cerebral computed tomography; CSF, cerebrospinal fluid; ICH, intracerebral hemorrhage; IVH, intraventricular hemorrhage.

The last CCT scan before the neurosurgical intervention was used to assess neuroradiological parameters including hydrocephalus defined by the Evans’ Index, midline shift defined as a deviation of the septum pellucidum from the falx cerebri, the anatomical location of ICH and the volumes of ICH, intraventricular hemorrhage (IVH), and intraventricular CSF (Figure 1). Volumes were measured by using the Brainlab SmartBrush software (Brainlab AG, Munich, Germany).

We classified the location of the ICH in lobar (frontal, central, temporal, occipital, and parietal), deep (basal ganglia, and thalamic), infratentorial (cerebellar, pons, and mesencephalic), and purely intraventricular.

### Statistical analysis

Statistical analyses were performed using SPSS software version 28.0 (IBM, Armonk, NY, USA). Categorical data were presented as counts and percentages, and continuous parameters as median and range. The chi-square and Mann–Whitney U tests were performed as statistically appropriate. Significant differences between nominal or ordinal variables were expressed as odds ratio (OR) and their 95% confidence intervals (95% CI). To investigate the predictive power of ICH, IVH, and intraventricular CSF volumes for the prediction of shunt implantation, binary regression models were conducted and the derived prediction formulas were tested using ROC models to define the most meaningful cutoff values according to sensitivity and specificity values. A value of *p* of <0.05 was considered statistically significant for all performed tests.

## Results

Between 2008 and 2021, a total of 120 patients underwent at least one neurosurgical procedure for a spontaneous ICH. After excluding patients with insufficient neuroradiological data, 114 out of 120 patients were analyzed (Table 1). At the time of hemorrhage, the median age was 61 (ranging 25–89) years and patients presented with a median GCS of 6 (ranging 3–15). Most ICHs were classified as deep location (66/114, 58%), with intraventricular hemorrhage being present in 82 out of 114 (72%) patients. In 70 out of 114 (61%) patients, a median midline shift of 8 mm (ranging 4–20 mm) was evident. Surgeries for clot removal alone, EVD insertion alone, or EVD insertion with clot removal were performed in 44 out of 114 (39%), 46 out of 114 (40%), and 24 out of 114 (21%) patients, respectively. In none of our patients’ tissue, plasminogen activator was applied intraventricularly.

**Table 1:** Patients after spontaneous ICH with and without acute hydrocephalus or EVD.

Variable	Overall (*n* = 114)	Hydrocephalus (*n* = 41)	No-hydrocephalus (*n* = 73)	Value of *p*	EVD (*n* = 70)	No-EVD (*n* = 44)	Value of *p*
Sex, male	57 (50%)	23 (56%)	34 (47%)	0.44	35 (50%)	22 (50%)	1.00
Median age at hemorrhage—in years	61 (25–89)	63 (25–89)	58 (31–80)	0.14	62 (25–89)	58 (35–80)	0.22
Median GCS at presentation	6 (3–15)	5 (3–14)	7 (3–15)	0.48	5 (3–15)	8 (3–15)	**0.05**
IVH	82 (72%)	35 (85%)	47 (64%)	**0.02**	61 (87%)	21 (48%)	**<0.001**
Median IVHS	15 (0–24)	18 (0–24)	15 (0–24)	**0.03**	18 (0–24)	9 (0–21)	**<0.001**
Location of hemorrhage							
Lobar	26 (23%)	3 (7%)	23 (31%)	**0.003**	6 (9%)	20 (46%)	**<0.001**
Frontal	8 (7%)	2 (5%)	6 (8%)	0.71	4 (6%)	4 (9%)	0.71
Central	7 (6%)	1 (2%)	6 (8%)	0.42	1 (1%)	6 (14%)	**0.01**
Temporal	3 (3%)	–	3 (4%)	–	–	3 (7%)	–
Occipital	5 (4%)	–	5 (7%)	–	1 (1%)	4 (9%)	0.07
Parietal	3 (3%)	–	3 (4%)	–	–	3 (7%)	–
Deep location	66 (58%)	24 (59%)	42 (57%)	1.00	45 (65%)	21 (48%)	0.12
Basal ganglia	47 (41%)	14 (34%)	33 (45%)	0.32	27 (39%)	20 (46%)	0.56
Thalamic	19 (17%)	10 (25%)	9 (12%)	0.12	18 (26%)	1 (2%)	**<0.001**
Infratentorial	19 (17%)	11 (27%)	8 (12%)	**0.04**	16 (23%)	3 (7%)	**0.04**
Cerebellar	17 (15%)	10 (25%)	7 (10%)	0.53	14 (20%)	3 (7%)	0.06
Pons	1 (1%)	–	1 (2%)	–	1 (1%)	–	–
Mesencephalic	1 (1%)	1 (2%)	–	–	1 (1%)	–	–
Purely IVH	3 (3%)	3 (7%)	–	–	3 (4%)	–	–
Comorbidities							
Hypertension	91 (80%)	35 (85%)	56 (77%)	0.34	57 (81%)	34 (77%)	0.64
Atrial fibrillation	17 (15%)	7 (17%)	10 (14%)	0.76	11 (16%)	6 (14%)	1.00
Diabetes mellitus	19 (17%)	14 (34%)	5 (7%)	**<0.001**	15 (21%)	4 (9%)	0.12
Anticoagulation	21 (18%)	7 (17%)	14 (19%)	1.00	13 (19%)	8 (18%)	1.00
Volumetry data							
IVH, all ventricles—median, in cm^3^	7.5 (0–99.3)	24 (0–99.3)	3.6 (0–80.2)	**<0.001**	22.1 (0–99.3)	0 (0–20.8)	**<0.001**
ICH, parenchyma—median, in cm^3^	32.8 (0–100)	18.8 (0–90.9)	43.8 (1–100)	**<0.001**	21.1 (0–97.2)	49.5 (6.1–100)	**<0.001**
CSF volume—median, in cm^3^	29.4 (2–205.2)	47.3 (11.7–205.2)	22.8 (2–83.1)	**<0.001**	40.3 (2–205.2)	20.5 (4.1–75.2)	**<0.001**

Values are expressed as numbers (%) or as median (range). Fisher’s exact test was used to perform group comparisons of categorical variables. Metric variables were analyzed by the Mann–Whitney *U*-test. CSF, cerebrospinal fluid; EVD, external ventricular drain; GCS, Glasgow coma scale; ICH, intracerebral hemorrhage; IVH, intraventricular hemorrhage; IVHS, intraventricular hemorrhage score. Statistically significant values are marked bold.

### Hydrocephalus at presentation

Hydrocephalus at presentation, measured by the Evans’ Index, occurred in 41 out of 114 (36%) patients. When comparing patients presenting with or without hydrocephalus, no significant differences in sex (*p* = 0.44), age (*p* = 0.14), and GCS (*p* = 0.48) could be observed (Table 1). Hydrocephalus occurred significantly more often in patients with infratentorial hemorrhage (*p* = 0.037) with an OR of 3 (1.1–8.2). The presence of IVH (*p* = 0.02) and DM2 (*p* < 0.001) was associated with acute hydrocephalus with an OR of 3.2 (1.2–8.7) and 7.1 (2.3–21.5), respectively. Furthermore, patients with hydrocephalus showed significantly higher IVH (*p* < 0.001) and CSF (*p* < 0.001) volumes and significantly lower ICH volumes (*p* < 0.001, Table 1) compared to patients without hydrocephalus.

### EVD treatment

In our study population, 70 out of 114 (57%) patients received an EVD. Between patients with and without an EVD, no statistically significant differences concerning sex (*p* = 1.00) and age (*p* = 0.22) were seen (Table 1).

The EVD placement was significantly more often performed in patients with a lower median GCS at presentation (*p* < 0.05), signs of hydrocephalus at presentation (*p* < 0.001, OR = 16.2, 4.6–57.4), IVH (*p* < 0.001, OR = 7.4, 3–18.6), thalamic ICH (*p* < 0.001, OR = 14.9, 1.9–116.1), infratentorial ICH (*p* = 0.037, OR = 4.1, 1.1–14.8), higher IVH (*p* < 0.001) and CSF volume (*p* < 0.001), lower ICH volume (*p* < 0.001) and with a higher IVHS (*p* < 0.001), as shown in Table 1. In patients with an EVD, the median volume of IVH, ICH, and intraventricular CSF was 22.1 cm^3^ (ranging 0–99.3 cm^3^), 21.1 cm^3^ (0–97.2 cm^3^), and 40.3 cm^3^ (2–205.2 cm^3^), respectively.

There was no statistically significant difference in the median total hemorrhage volume (IVH + ICH) in patients with an EVD (52.4 cm^3^, ranging 1–135.2 cm^3^) compared to patients without an EVD treatment (51.7 cm^3^, ranging 6.1–110.1 cm^3^, *p* = 0.93). However, the median ratio between the total hemorrhage volume and intraventricular CSF was significantly lower in patients with an EVD (1.1, ranging 0–63.3) compared to patients who did not receive an EVD (2.7, ranging 0.3–14.2, *p* < 0.001).

In 21 out of 70 (30%) patients, EVD placement was performed bilaterally. Patients who received an EVD bilaterally had a significantly higher median IVH (38.9 cm^3^, ranging 3.3–99.3 cm^3^ vs. 17.5 cm^3^, ranging 0–83.1 cm^3^; *p* < 0.001) and lower ICH volume (8.4 cm^3^, ranging 0–47.1 cm^3^ vs. 23.9 cm^3^, ranging 1–97.2 cm^3^, *p* < 0.001) compared to patients after a unilateral EVD placement. Moreover, patients received significantly more often bilateral EVD after a deep hemorrhage (18/21, 86%, *p* = 0.016), thalamic ICH (10/21, 48%, *p* = 0.015), and purely IVH (3/21, 14%, *p* = 0.024).

### Shunt treatment

After excluding patients, who died within 30 days (*n* = 18/120, 15%), 99 out of 120 patients could be evaluated for shunt dependency (Table 2). Of those, 57 (58%) patients underwent treatment with an EVD. Shunt implantation was performed in 11 out of 99 (11%) patients. The median time of shunt implantation after admission was 30 days (ranging 16–75 days). There was no statistically significant difference in regard to shunt placement between patients after EVD insertion alone (9 out of 36 patients) and EVD insertion with clot removal (2 out of 21 patients, *p* = 0.19). Between patients with and without a shunt in place, no statistically significant differences concerning sex (*p* = 0.53), age (*p* = 0.53), GCS at presentation (*p* = 0.59), and IVH (*p* = 0.50) was encountered (Table 2).

**Table 2:** Patients after spontaneous ICH with and without shunt.

Variable	Overall (*n* = 99)	Shunt (*n* = 11)	No-shunt (*n* = 88)	Value of *p*
Sex, male	48 (49%)	4 (36%)	44 (50%)	0.53
Median age at hemorrhage—in years	61 (25–89)	63 (25–74)	61 (32–89)	0.53
Median GCS at presentation	7 (3–15)	4 (3–14)	7 (3–15)	0.59
IVH	70 (71%)	9 (82%)	61 (69%)	0.50
Median IVHS	15 (0–24)	15 (8–18)	15 (0–24)	0.93
Location of hemorrhage				
Lobar	25 (25%)	–	25 (28%)	0.06
Frontal	8 (8%)	–	8 (9%)	–
Central	7 (7%)	–	7 (8%)	–
Temporal	3 (3%)	–	3 (3%)	–
Occipital	4 (4%)	–	4 (5%)	–
Parietal	3 (3%)	–	3 (3%)	–
Deep location	56 (57%)	6 (55%)	50 (57%)	1.00
Basal ganglia	38 (39%)	1 (9%)	37 (42%)	**0.05**
Thalamic	18 (18%)	5 (46%)	13 (15%)	**0.03**
Infratentorial	15 (15%)	4 (36%)	11 (12%)	0.06
Cerebellar	13 (13%)	4 (36%)	9 (10%)	**0.04**
Pons	1 (1%)	–	1 (1%)	–
Mesencephalic	1 (1%)	–	1 (1%)	–
Purely IVH	3 (3%)	1 (9%)	2 (3%)	0.30
Comorbidities				
Hypertension	80 (81%)	8 (73%)	72 (82%)	0.44
Atrial fibrillation	15 (15%)	2 (18%)	13 (15%)	0.67
Diabetes mellitus	15 (15%)	2 (18%)	13 (15%)	0.67
Anticoagulation	17 (17%)	1 (9%)	16 (18%)	0.68
Volumetry data				
Acute hydrocephalus	35 (35%)	9 (82%)	26 (30%)	**<0.001**
IVH, all ventricles—median, in cm^3^	6.3 (0–99.3)	25.2 (0–51.4)	4.9 (0–99.3)	0.07
ICH, parenchyma—median, in cm^3^	32.2 (0–100)	21 (0–53.3)	37.7 (0–100)	**0.03**
CSF volume—median, in cm^3^	26.8 (2–205.2)	61.5 (28.9–205.2)	24.4 (2–109.5)	**<0.001**

Values are expressed as numbers (%) or as median (range). Fisher’s exact test was used to perform group comparisons of categorical variables. Metric variables were analyzed by the Mann–Whitney *U*-test. CSF, cerebrospinal fluid; GCS, Glasgow coma scale; ICH, intracerebral hemorrhage; IVH, intraventricular hemorrhage; IVHS, intraventricular hemorrhage score. Statistically significant values are marked bold.

In shunted patients, the median volume of IVH, ICH, and intraventricular CSF was 25.2 cm^3^ (ranging 0–51.4 cm^3^), 21 cm^3^ (0–53.3 cm^3^), and 61.5 cm^3^ (28.9–205.2 cm^3^), respectively. Shunt implantation was significantly more often performed in patients with hydrocephalus at presentation (*p* < 0.001, OR = 10.7, 2.2–53.1), thalamic ICH (*p* = 0.026, OR = 4.8, 1.3–18.1), cerebellar ICH (*p* = 0.036, OR = 5.02, 1.2–20.5), lower ICH volume (*p* = 0.03), higher CSF volume (*p* < 0.001), and a higher IVH distribution in the third ventricle (*p* = 0.03), as shown in Tables 2, 3. Furthermore, higher IVH volumes (25.2 cm^3^, ranging 0–51.4 cm^3^) revealed no significant difference between patients needing a CSF shunt and those without (4.9 cm^3^, ranging 0–99.3 cm^3^, *p* = 0.07).

**Table 3:** Blood distribution between ventricles in patients with and without shunts.

Variable	Overall (*n* = 99)	Shunt (*n* = 11)	No-shunt (*n* = 88)	Value of *p*
I. Ventricle				0.26
No blood or small amount of layering	46 (47%)	3 (27%)	43 (49%)	
Up to one-third filled with blood	30 (30%)	6 (55%)	24 (27%)	
One to two-thirds filled with blood	15 (15%)	2 (18%)	13 (15%)	
Mostly or completely filled with blood	8 (8%)	0	8 (9%)	
II. Ventricle				0.23
No blood or small amount of layering	43 (44%)	3 (27%)	40 (46%)	
Up to one-third filled with blood	27 (27%)	6 (55%)	21 (24%)	
One to two-thirds filled with blood	20 (20%)	2 (18%)	18 (20%)	
Mostly or completely filled with blood	9 (9%)	0	9 (10%)	
III. Ventricle				**0.03**
No blood or small amount of layering	43 (43%)	2 (18%)	41 (47%)	
Up to one-third filled with blood	2 (2%)	0 (0)	2 (2%)	
One to two-thirds filled with blood	3 (3%)	2 (18%)	1 (1%)	
Mostly or completely filled with blood	51 (52%)	7 (64%)	44 (50%)	
IV. Ventricle				0.6
No blood or small amount of layering	49 (50%)	4 (36%)	45 (51%)	
Up to one-third filled with blood	2 (2%)	0 (0)	2 (2%)	
One to two-thirds filled with blood	2 (2%)	0 (0)	2 (2%)	
Mostly or completely filled with blood	46 (46%)	7 (64%)	39 (45%)	

Values are expressed as numbers (%) or as median (range). Fisher–Freeman–Halton’s exact test was used to perform group comparisons of categorical variables. Statistically significant values are marked bold.

There was no statistically significant difference in the median total hemorrhage volume (IVH + ICH) in shunted patients (39.6 cm^3^, range: 12.2–83.2 cm^3^), compared to patients without needing a CSF shunt (50.7 cm^3^, range: 1–135.2 cm^3^, *p* = 0.328). However, the ratio between the total hemorrhage volume and intraventricular CSF was significantly lower in shunted patients (0.6, range: 0.1–2.3) compared to patients without a CSF shunt (2.2, range: 0–63.3, *p* = 0.005).

### Differences in EVD treatment between patients with and without a shunt

No patient without an EVD in the acute phase developed a secondary hydrocephalus necessitating the implantation of a CSF shunt. Differences in EVD treatment duration or the amount of drained CSF per day between patients with and without a CSF shunt were analyzed in 51 out of 57 (90%) patients due to insufficient data in the remaining six patients. There was no significant difference in the median amount of drained CSF/day between shunted (140 mL, ranging 66–224 mL) and non-shunted patients (118 mL, ranging 0–239 mL, *p* = 0.330). However, shunted patients had a significantly longer median EVD treatment duration (25 days, ranging10–46 days) compared to non-shunted patients (14 days, ranging 1–66 days, *p* = 0.002). Furthermore, there was no significant difference in revision surgeries for an EVD placement between patients with (*n* = 7 out of 10, 70%) and w/o a shunt (*n* = 14 out of 41, 34%; *p* = 0.07).

### Prediction of shunt dependency

To predict later shunt dependency, we generated a logistic regression model including IVH, ICH, and intraventricular CSF based on all 99 patients and established the following formula:


$$-0.005^{*}ICH+0.029^{*}IVH+0.036^{*}\mathrm{intraventricular}\;CSF$$

**Figure 2:**
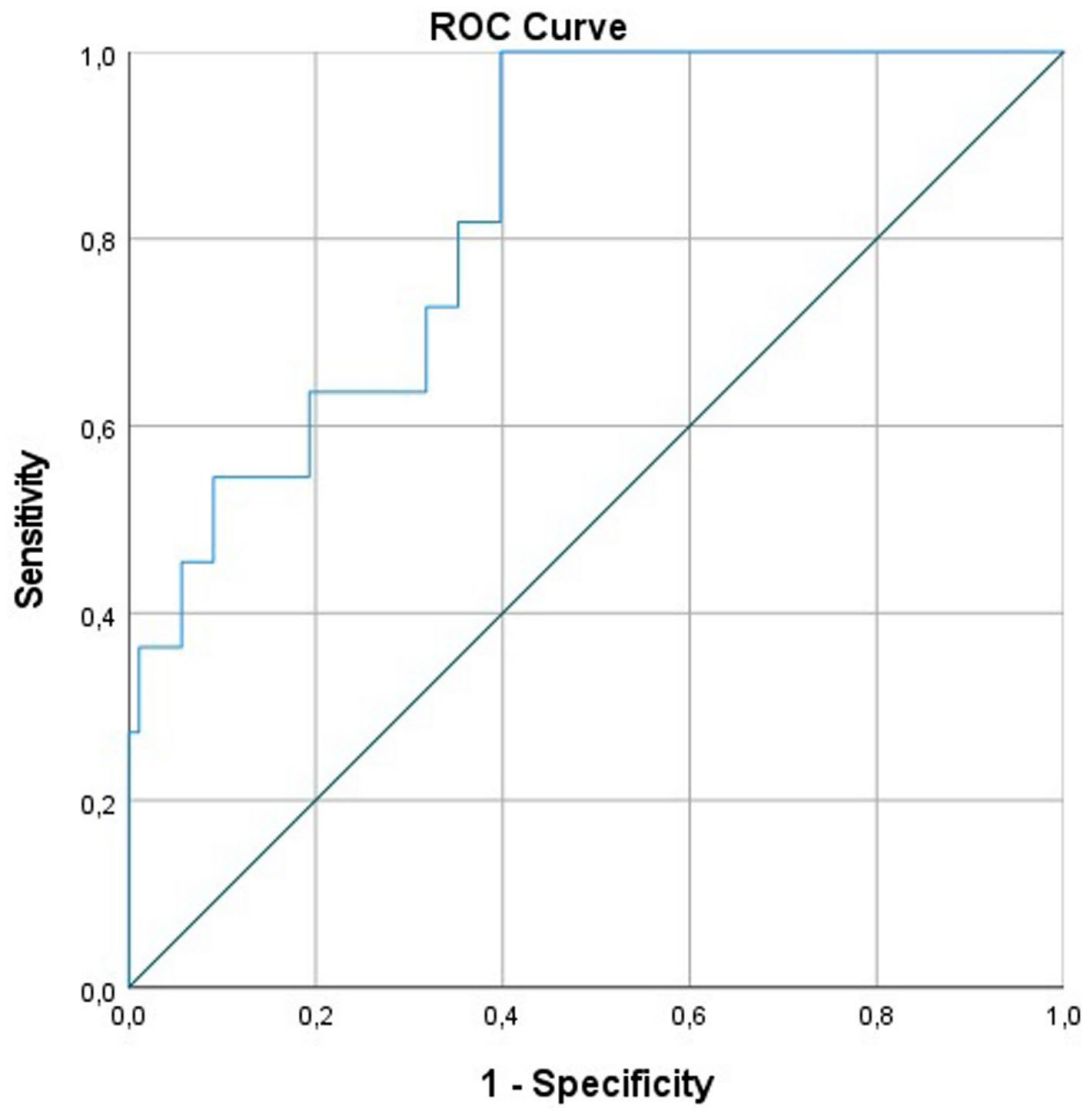
ROC curve for the prediction of shunt implantation according to ICH, IVH, and intraventricular CSF. CSF, cerebrospinal fluid; ICH, intracerebral hemorrhage; IVH, intraventricular hemorrhage; ROC, receiver operating characteristics.

The ROC model demonstrated a sensitivity of 82% and a specificity of 65% to predict the necessity for a shunt at a cutoff value of 1.9 with an AUC of 0.835 (Figure 2).

## Discussion

In this study, we could elaborate that (1) a lower ratio between the total hemorrhage and intraventricular CSF volumes (*p* = 0.007), (2) hydrocephalus (*p* < 0.001) at presentation defined by the Evans’ Index as well as an increased intraventricular CSF volume (*p* < 0.001), (3) thalamic (*p* = 0.026) and cerebellar ICH (*p* = 0.036), and (4) a higher IVH distribution in the third ventricle was associated with shunt dependency. This is in contrast to many previous reports and can be discussed under various aspects (6, 7, 9).

First, the definition of hydrocephalus in this specific patient cohort has been very heterogeneous, which is at least partly reflected by the high range in the reported incidence of 8.9–31% (6, 10, 18). While various hydrocephalus scores have been implemented over the past decades for different pathological substrates, the generalization of these scores and their application for patients with spontaneous ICH and IVH is difficult (19–21). The bicaudate index, for example, was first introduced by Barr et al. as a tool to assess Huntington diseases and brain atrophy on CCT scans (19). Subsequently, it became a commonly used measure for the diagnosis of hydrocephalus in various neurosurgical patient cohorts, especially in patients with subarachnoid hemorrhage (22–25). In contrast, the Evans’ Index has been widely used as a neuroradiological additive to aid in the diagnosis of normal pressure hydrocephalus, and more recently has been assessed in combination with volumetric measurements (26–29).

Apart from the fact that the interobserver variability of measuring these scores limits their applicability, all of these scores were not intended to measure hydrocephalus in the setting of space-occupying bleeding with or w/o intraventricular hemorrhage (30, 31). To overcome this limitation, we aimed to focus on more objective and independent factors. The intraventricular CSF volume in our patient cohort was revealed to be significantly different between patients who needed a CSF-shunt implantation compared to patients who did not (*p* < 0.001). The higher the volume of the intraventricular CSF at presentation, the higher the likelihood that the patients received a shunt after the acute phase. In the same context and somewhat counterintuitively, the volume of the IVH did not reach significance to increase the likelihood of the need for a shunt implantation (9).

Second, similar discordance between our results and those given in the literature exists regarding the volume of the ICH and its influence on the subsequent need for shunt implantation. While we observed that ICH volumes were significantly lower in shunted patients compared to non-shunted patients (*p* = 0.03), others did not (6, 7).

However, former studies have computed ICH volumes by using the simple ABC/2 method only, which measures the volume of a three-dimensional lesion with A being the greatest hemorrhage diameter, B the diameter 90° to A, and C the approximate number of CT slices multiplied by the slice thickness (6, 10, 32–34). While its clinical application seems easy and straightforward, the inhomogeneous pattern and complex three-dimensional nature of ICH leads to inaccurate and unspecific results also limiting the possibility to compare different cohorts. Only one study so far has used specific software in order to accurately quantify IVH volumes (9). In this study, Kuo et al. showed that the bicaudate index, hemorrhage volume in lateral ventricles, hemorrhage volume in the fourth ventricle, and the ratio of hemorrhage volume in lateral ventricles to that in third and fourth ventricles were predictors for shunt dependency (9).

Third, the location of ICH and the distribution of IVH and their impact on later shunt dependency have generated conflicting results. While our study corroborates with the studies by Zacharia et al. and Miller et al. showing that a thalamic location of the ICH is associated with later shunt dependency, others did not (6, 7, 35). Discrepancies might be explained by a different combination of factors including the location of ICH within the thalamus, site of ventricular rupture into the ventricles, i.e., third or lateral ventricle, and volumes of ICH and IVH. While it seems plausible to us that a thalamic ICH with rupture into the third ventricle and obstruction of the Foramen of Monro, and aqueduct be the cause for acute obstructive hydrocephalus, the impact on the development of chronic hydrocephalus is less clear (7). Provided that the chronic obstruction of the arachnoid villi, Foramen of Monro, and aqueduct may be the cause of chronic CSF circulation disturbances, one could assume that the amount of the IVH volume would be predictive for shunt dependency (7, 8, 35). However, so far, no study has shown an association between IVH volume and shunt dependency.

As all three aforementioned metric variables have been shown to influence the development of a chronic CSF circulation disturbance and the need for a shunt implantation, we thought to take all of them into account and generated a formula to predict shunt dependency from the CCT scan at presentation. Our ROC model demonstrated a sensitivity of 82% and a specificity of 62% for the necessity of shunt implantation at a cutoff value of 1.9 with an AUC of 0.835. While this model seems promising in supporting physicians in the treatment and care of these patients, future studies are needed to evaluate the efficacy of this prediction model prospectively.

Finally, we aimed to elaborate if the amount of daily CSF output has an influence on later shunt dependency as suggested in the CLEAR III trial (35). However, we could not find any correlation between the amount of daily CSF output and shunt dependency. This discrepancy might be explained by differences in the EVD management and weaning strategies between centers.

## Limitations

Apart from all the inherent limitations of a retrospective study, one limitation is that only patients with spontaneous ICH who underwent a neurosurgical treatment have been included. This could explain the varying ICH and IVH volumes measured in our cohort compared to previous studies. Furthermore, our prediction formula is limited by the fact that we only used neuroradiological variables in predicting shunt dependency. We believe that the inclusion of clinical variables and CSF parameters may improve the validity of such a prediction tool, which needs to be evaluated in a systematic prospective manner.

## Conclusion

Predicting the need for a CSF shunt in patients with spontaneous ICH remains a challenging task, but our results add to the current understanding of the contributing factors. We elaborated a prediction tool that needs further prospective evaluation but has the potential to help clinicians guiding their treatment and care of these patients.

## Data availability statement

The original contributions presented in the study are included in the article/supplementary material, further inquiries can be directed to the corresponding author.

## Ethics statement

The study involving humans was approved by Ethics Committee of the Medical University of Vienna (EK 1055/2022). The studies were conducted in accordance with the local legislation and institutional requirements. Written informed consent for participation was not required from the participants or the participants’ legal guardians/next of kin in accordance with the national legislation and institutional requirements.

## Author contributions

FK: Conceptualization, Formal analysis, Investigation, Methodology, Visualization, Writing – original draft, Writing – review & editing. VZ: Investigation, Writing – review & editing. AC: Formal analysis, Investigation, Writing – review & editing. SS: Investigation, Writing – review & editing. AR: Investigation, Writing – review & editing. JH: Investigation, Writing – review & editing. KR: Investigation, Writing – review & editing. CD: Conceptualization, Investigation, Methodology, Supervision, Writing – original draft, Writing – review & editing.
